# Modified auxin improves the auxin-inducible degradation (AID) system for laid *C. elegans* embryos

**DOI:** 10.17912/micropub.biology.000190

**Published:** 2019-12-10

**Authors:** Takefumi Negishi, Masayo Asakawa, Masato T Kanemaki, Hitoshi Sawa

**Affiliations:** 1 Multicellular Organization Laboratory, National Institute of Genetics, 1111 Yata, Mishima, Shizuoka 411-8540, Japan; 2 Molecular Cell Engineering Laboratory, National Institute of Genetics, 1111 Yata, Mishima, Shizuoka 411-8540, Japan; 3 Department of Genetics, School of Life Science, SOKENDAI (The Graduate University for Advanced Studies), 1111 Yata, Mishima, Shizuoka 411-8540, Japan

**Figure 1.**
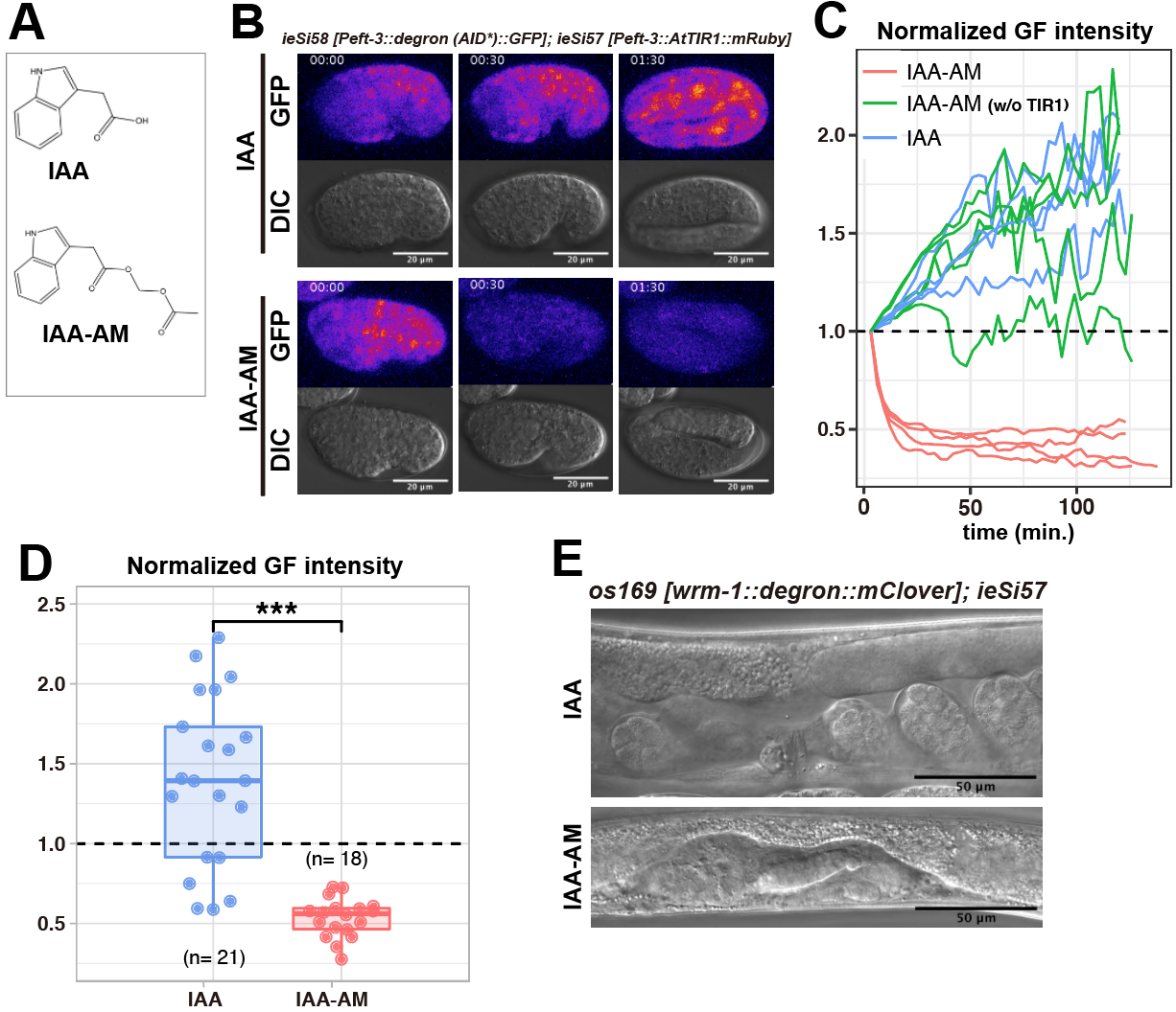
(A) Chemical structural formula of indole-3-acetic acid (IAA) and acetoxymethyl indole-3-acetic acid (IAA-AM). (B) Frames from time lapse videos of *ieSi58* [P_eft-3_::degron (AID*)::GFP]; *ieSi57* [*P_eft_–_3_::AtTIR1::mRuby*] laid embryos in IAA (1 mM) or IAA-AM (1 mM); elapsed time (hours:minutes) from the beginning of recording is indicated. GFP panels show maximum intensity projection images, and green fluorescent signal is colored with “Fire” look-up-table. Scale bar, 20 μm. (C) Plots of green fluorescent (GF) intensity in timelapse observation. Red lines: IAA-AM treatment of *ieSi58 ieSi57* embryos, green lines: IAA-AM treatment of *ieSi58* embryos (no TIR1 present), blue lines: IAA treatment of *ieSi58 ieSi57* embryos. Mean values of GF intensity in eggshells were obtained every three minutes, and normalized by the intensity at 0 min. (D) Comparison of GF depletion between IAA and IAA-AM treatment of *ieSi58; ieSi57* embryos. Mean values of GF intensity within eggshells were obtained after each treatment (two hours), and normalized with that of the beginning, *** indicates p < 0.01, p= 1.6 x 10^-8 ^(Wilcoxon rank sum test). (E) Representative images of *os169 ieSi57* adults, which are IAA or IAA-AM treated during embryos development (two hours).

## Description

The targeted protein degradation systems in which a protein accompanying with specific tags can be degraded are developed as an approach of conditional loss of function analyses (Natsume and Kanemaki 2017). The insertion of tags into the gene loci by the CRISPR/Cas9 system allows us to deplete endogenous proteins in stage and cell specific manners. So far, four systems can be used to deplete the tagged proteins in *C . elegans* (Armenti et al. 2014; Zhang et al. 2015; Wang et al. 2017; Wu et al. 2017). The auxin-inducible degradation (AID) system can degrade the tagged protein by administration of the phytohormone auxin. In the AID system, a plant-derived TIR1 F-box protein can form an E3 ubiquitin ligase complex with the endogenous Skp and Cullin proteins, and in the presence of auxin, the complex interacts with a degron tag derived from the IAA17 transcriptional repressor (Nishimura et al. 2009; Yesbolatova et al. 2019). Consequently, the degron-tagged proteins are polyubiquitinated for degradation by the proteasome. With the controlled expression of TIR1 and administration of auxin, the AID system can allow us to perform spaciotemporal protein depletion. Although this system can be a powerful tool for conditional loss of function analyses, it appears to be difficult to degrade proteins in embryos, especially at late embryonic stages, since efficiencies of degradation in laid embryos surrounded by the eggshell is low compared to those in hatched larvae or adults (Zhang et al. 2015).

Here, we report that acetoxymethyl indole-3-acetic acid (IAA-AM), a cell permeable analog of natural auxin IAA (Figure1A), is more effective in triggering the degradation of tagged proteins in embryos. To test the efficiency of protein degradation, we used the previously reported strain ubiquitously expressing both a 44-amino acid (-aa) degron derived from the *Arabidopsis* IAA17 protein (known as AID*) fused with GFP and *Arabidopsis* TIR1 (AtTIR1) fused with mRuby (*ieSi58* [P_eft-3_::degron (AID*)::GFP] and *ieSi57* [*P_eft_–_3_::AtTIR1::mRuby*])(Morawska and Ulrich 2013; Zhang et al. 2015). When we put laid embryos into the drop of M9 buffer containing 1 mM IAA-AM (AM form of IAA), we observed rapid decrease of green fluorescence (GF) intensity (Figure1B, C and D). In contrast to IAA-AM, the embryos treated with IAA showed increased GF intensity due to elevated expression of degron-tagged GFP (Figure1B, C and D). We also confirmed that IAA-AM did not cause the decreased GF intensity without the *AtTIR1* transgene (Figure1C).

Next, we tried to deplete the endogenous protein in laid embryos. WRM-1 is the *C. elegans* homolog of β-catenin and is essential for asymmetry of most cell divisions (Mizumoto and Sawa 2007). Since WRM-1 is required for asymmetric division of EMS in the 4-cell stage embryo (Rocheleau et al. 1997), conditional knockdown approaches are required for the investigation of its function in later stages. To test whether the AID system with IAA-AM allows us to perform conditional knockdown of *wrm-1* in embryos, we inserted a fusion tag composed of the mini-auxin inducible degron (mAID), a 68-aa degron derived from the *Arabidopsis* IAA17 protein that showed similar degradation efficiency with that of AID* (Li et al. 2019), and mClover at the 3’ end of *wrm-1* endogenous locus by the CRISPR/Cas9 system (Natsume et al 2016) (Dickinson et al. 2015). We put laid embryos of the strain carrying *wrm-1::mAID::mClover (os169)* and *P_eft_-3::AtTIR1::mRuby (ieSi57)* into drops of 1 mM IAA-AM or IAA (both with 0.2% DMSO in M9 buffer) for two hours. After washing with M9 buffer two times, we placed embryos on NGM plates without the inducing ligand. At the young adult stage, we scored elongation of gonadal arms guided by the distal tip cells (DTCs) whose production requires *wrm-1*-regulated asymmetric divisions (Siegfried et al. 2004). When we treated *os169 ieSi57* embryos with 1 mM IAA-AM, all animals (13/13) shows no gonadal extension. In contrast, treating with 1 mM IAA caused this phenotype only in 2/17 animals (15/17 showed normal gonad) (Figure 1E). In addition, all *os169 ieSi57* embryos treated with 0.2% DMSO (16/16) or *ieSi58 ieSi57* embryos treated with 1mM IAA-AM (19/19) produced animals with normal gonads. These results show that the AM modification of IAA induces efficient protein degradation in laid embryos with the AID system. Since the AM modification is widely used to improve cell membrane permeability (Schultz 2003), IAA-AM is likely to penetrate into the eggshell more easily than IAA. After penetration into cells, endogenous cytoplasmic esterases are known to cleave the AM bond, making the compound again impermeant to cell membrane (Schultz 2003). We note that the chemical cleavage reaction of the AM bond produces formaldehyde (Schultz 2003), which may be toxic to cells at high concentrations. In this study, however, we found the application of IAA-AM at a concentration of 1 mM for two hours allowed normal development including gonad elongation. Our data presented here show IAA-AM will broaden the possible applications of the AID system for conditional loss of function analyses in *C. elegans*.

## Reagents

**Plasmid construction**: For insertion of degron tag and mClover into 3’ end of *wrm-1* locus by CRISPR/Cas9 system, the fragment of mAID::mClover was amplified with the primers (F: 5’-GCCTCAGGAGCATCGGGATCCGGTGCAGGCGCCAAGG-3’, R: 5’- AAAGTACAGATTCTCCTTGTACAGCTCGTCCATGCCA-3’) from pMK290 (Addgene: #72828). The fragment of self-excising cassette (SEC) described in Dickinson et al., 2015 was amplified with the primers (F: 5’-CGATGCTCCTGAGGCTCCCGAT-3’, R: 5’- GAGAATCTGTACTTTCAATCCG-3’) from pDD287 (Addgene #70685). These fragments were assembled by InFusion HD (Takara Clontech). Then, 5’ and 3’ *wrm-1* homology arms were amplified from *C. elegans* genome with the following primers:

wrm-1RightL: 5’-AGCGAGGAAGACTTGTGAATGAATCTTTGTGCGGGTA-3’,

wrm-1RightR: 5’-CTATGACCATGTTATAACTGGTGGTGATCGTGCTTGG-3’

wrm-1LeftL: 5’-AACGACGGCCAGTCGTTTTGTTGAATGCAAATATGTG-3’

wrm-1LeftR: 5’-GGATCCCGATGCTCcCATTAGTTGTCGATGATGCTGC-3’.

Both fragments of homology arms were assembled into the backbone and SEC fragments amplified from the above-described construct with the primers (backbone-F: 5’-CGACTGGCCGTCGTTTTACAAC-3’, backbone-R: 5’- ATAACATGGTCATAGCTGTTTC-3’ and SEC-F: 5’-GGAGCATCGGGATCCGGTGCAG-3’, SEC-R: 5’-CAAGTCTTCCTCGCTGATCAAC-3’) by InFusion HD (Takara Clontech). The vector expressing the guide RNA for *wrm-1* was constructed with the primers including the target sequence (F: 5′-[ATGTGAATGAATCTTTGTGC]GTTTTAGAGCTAGAAATAGCAA-3′, R: 5′-AAGATTCATTCACATAAACATTTAGATTTGCAATTCA-3′, brackets indicate the target sequence) by inverse PCR with PU6::unc-119_sgRNA (Addgene #46469).

**Strain construction**: All *C. elegans* strains were cultured by standard methods. Plasmids (50 ng/µl mAID::mClover with *wrm-1* homology arms construct, 50 ng/µl *wrm-1* guide RNA construct, 50 ng/µl *sur-5*::*GFP* co-injection marker and Cas9 expression construct (pDD162, Addgene #47549)) were microinjected into N2. After picking an animal displaying a roller phenotype, self-excising cassette was excised as described (Dickinson et al. 2015). Then, we crossed this strain to the *P_eft-3_::AtTIR1::mRuby* strain.

**Observation**: In time lapse observation, embryos were mounted on slides with 20 μm diameter polystyrene beads (Polyscience #18329) diluted 1:30 in M9 buffer containing 1 mM IAA or IAA-AM (stocks 500 mM in DMSO)(Bao and Murray 2011). In the endogenous protein degradation experiments, laid embryos were put in drops of M9 buffer containing 0.2% DMSO, 1 mM IAA or IAA-AM for two hours, and transferred into M9 buffer two times before hatching. Washed embryos were allowed to develop on new NGM plates. All imaging was performed with Zeiss LSM700 microscope using a 63x N.A. 1.40 oil-immersion objective. Acquired images were processed by Fiji (Schindelin et al. 2012).

**Strains**:

CA1202: *ieSi57* (*P_eft_-3::AtTIR1::mRuby*) II ; *ieSi58* (P_eft-3_::degron (AID*)::GFP) IV, CGC

HS3280: *ieSi58* (P_eft-3_::degron (AID*)::GFP) IV , this study

HS3176: *ieSi57* (*P_eft_-3::AtTIR1::mRuby*) II; *wrm-1(os169* [*wrm-1::mAID::mClover*]) III, this study

**IAA and IAA-AM:**

IAA (indole-3-acetic acid) was purchased from Nacalai tesque (#19119-61). IAA-AM was custom synthesized by Tokyo Chemical Industry Co., Ltd. Both ligands were dissolved in DMSO to make a 500 mM stock solution before storing at -20˚C.
